# Precision Agriculture and Sensor Systems Applications in Colombia through 5G Networks

**DOI:** 10.3390/s22197295

**Published:** 2022-09-26

**Authors:** Wilson Arrubla-Hoyos, Adelaida Ojeda-Beltrán, Andrés Solano-Barliza, Geovanny Rambauth-Ibarra, Alexis Barrios-Ulloa, Dora Cama-Pinto, Francisco Manuel Arrabal-Campos, Juan Antonio Martínez-Lao, Alejandro Cama-Pinto, Francisco Manzano-Agugliaro

**Affiliations:** 1 Faculty of Engineering, Bicentennial Scholarship–Ministry of Sciences, Universidad Nacional Abierta y a Distancia, Sincelejo 700002, Colombia; 2 Faculty of Economy, Bicentennial Scholarship–Ministry of Sciences, Universidad del Atlántico, Puerto Colombia 081007, Colombia; 3 Faculty of Engineering, Bicentennial Scholarship–Ministry of Sciences, Universidad de La Guajira, Riohacha 440002, Colombia; 4 Department of Electronics Engineering, Faculty of Engineering, Bicentennial Scholarship–Ministry of Sciences, Universidad de Sucre, Sincelejo 700001, Colombia or; 5 Department of Computer Architecture and Technology, University of Granada, 18071 Granada, Spain; 6 Department Engineering, University of Almeria, 04120 Almería, Spain; 7 Department of Computer Science and Electronics, Universidad de la Costa, Barranquilla 080002, Colombia

**Keywords:** 5G, agriculture, Colombia, smart farm, spectrum, sustainability

## Abstract

The growing global demand for food and the environmental impact caused by agriculture have made this activity increasingly dependent on electronics, information technology, and telecommunications technologies. In Colombia, agriculture is of great importance not only as a commercial activity, but also as a source of food and employment. However, the concept of smart agriculture has not been widely applied in this country, resulting in the high production of various types of crops due to the planting of large areas of land, rather than optimization of the processes involved in the activity. Due to its technical characteristics and the radio spectrum considered in its deployment, 5G can be seen as one of the technologies that could generate the greatest benefits for the Colombian agricultural sector, especially in the most remote rural areas, which currently lack mobile network coverage. This article provides an overview of the current 5G technology landscape in Colombia and presents examples of possible 5G/IoT applications that could be developed in Colombian fields. The results show that 5G could facilitate the implementation of the smart farm in Colombia, improving current production and efficiency. It is useful when designing 5G implementation plans and strategies, since it categorizes crops by regions and products. This is based on budget availability, population density, and regional development plans, among others.

## 1. Introduction

According to the Food and Agriculture Organization of the United Nations (FAO), agriculture “is a manmade activity to produce food that meets the needs and food security of the people of a given population” [1]. Agriculture is the main food source for the world’s population and represents an economic income for developing countries through its exports. Although it generates these benefits, current World Food Program estimates indicate that approximately 690 million people in the world suffer from hunger, forcing the United Nations (UN) to prioritize within the Sustainable Development Goals (SDGs) an increase in agricultural productivity and sustainable food production to alleviate the risk of hunger.

The inclusion of technologies could contribute to the fulfillment of SDG 2 “end hunger”, projected in the 2030 agenda by the UN through the strengthening of agricultural processes to meet the food needs of the world’s population. Some relevant technologies to contribute to this goal of strengthening agricultural processes are smart agriculture (SA), precision agriculture (PA), 5G, and the Internet of Things (IoT), which can be scaled up in developing countries such as Colombia to strengthen agriculture.

In Colombia, there are crops of great importance, not only for the food sustainability of its population (e.g., rice, cassava, avocado, and corn) but also for other activities such as ornamental flowers, or cassava, used as raw material in the production of starch. Many of these plantations cover large geographical areas or are located far from the main municipalities, making their control and management difficult. In this sense, future 5G deployments could become an excellent opportunity for Colombian growers to optimize their operations, from the initial planning stage of the plantation to the final commercialization of their production.

The main objective of this article is to project the possible applications of PA (precision agriculture) and SF (smart farming) in Colombia through 5G networks. In addition, an analysis is made of the potential applications of PA with IoT that could contribute to the development of agriculture and livestock in different Colombian regions, considering the specificities of the products in the geographic regions of the Colombian territory and its projection in the national and international market.

The specific objectives of this article are as follows:

- To contextualize the current situation of 5G network deployment in Colombia and its projection to enhance smart agriculture.
- To document smart agriculture applications with IoT and 5G in Colombian departments and regions.
- To propose future applications of 5G/IoT in the field of smart agriculture in Colombia.

### Related Work

Previous research has exposed the important synergies of 5G and IoT and their impact on agriculture. The authors of [2] conducted a study on 5G technology in the agricultural sector, analyzing the need and role of SF and PA. The authors of [3,4] showed the progress in the design and implementation of a sustainable greenhouse prototype supported by IoT applications connected to the 5G network.

Initiatives associated with the MERLIN project in India [5] combine artificial intelligence (AI) and wireless communication system based on 5G and IoT with a cloud-based supervised and semi-supervised database. This technology solution takes input data from each task it performs and uses it as feedback for further processing. In India [6], they also automate and improve productivity performance using IoT and remote sensors applied to agriculture.

The authors of [7] explained the improvement of agricultural management with the contribution of SF, showing the importance of using a 5G mobile network in the development of smart systems due to the high data transfer speed (up to 20 Gbps) and the ability to link many devices per square kilometer.

Data related to Agriculture 4.0 are shown below [8], comprising 5G usage scenarios, frequency bands, applications, the relationship with IoT, and the implementation of 5G deployment in Colombia.

The authors of [8] showed the interaction among 5G, Mobile Edge, and robotics in SF scenarios, based on Industry 4.0 principles for rural area scenarios.

## 2. Background

Definitions related to Agriculture 4.0, smart agriculture, 5G usage scenarios, frequency bands, applications, the relationship with IoT, and the implementation of 5G deployment in Colombia are presented below.

### 2.1. Agriculture 4.0 

**Figure 1:**
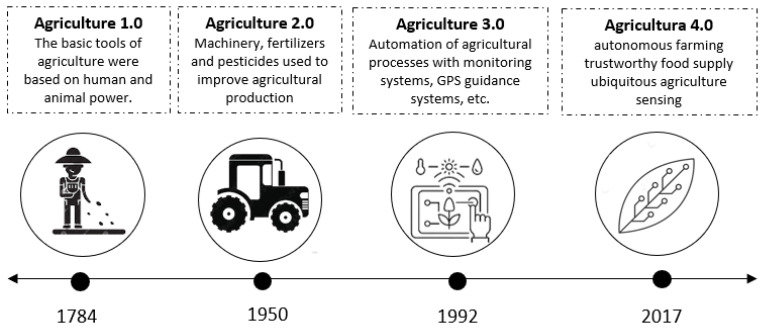
Timelines of agricultural revolutions (based on [10]).

Talking about Agriculture 4.0 or digital agriculture (DA) implies knowing its evolution through time (see Figure 1). The first agricultural methods used papyrus to develop irrigation systems such as those used by the Egyptians and Greeks around 6000 BC [9], which depended on human labor and animal power [10]. During the 17th century, the era of feudalism emerged on the European continent, including new agricultural treatment techniques, and the incursion of machinery for soil treatment, sowing, weeding, irrigation, and harvesting [7].

As agriculture is one of the most important economic pillars in the economy of countries, it needs to adopt new technologies such as PA, which apply inputs in the right place at the right time [11]. In this context, large economies around the world are applying PA to improve agricultural processes for products that contribute significantly to economic development. For example, China and India have been applying PA to increase tea crop yields for more than a decade. Similarly, in Malaysia, it is being used to optimize crop-specific fertilization techniques [12]. These successful experiences and others reported in the literature, provide a glimpse of some PA application scenarios that can serve as a reference for other countries where agriculture is important for the economy and food sustainability.

According to data from the Food and Agriculture Organization of the United Nations (FAO), in Colombia, between 20% and 40% of crops are lost due to little or no treatment of diseases and pests, resulting in losses of more than six million tons per year. The technological incursion in the Colombian agricultural sector has been progressive, with a predominance of artisanal methods, which of course have repercussions for the production of products with qualities that are not very competitive in international markets [13]. To achieve improvements in the quantity and quality of agricultural products, it is necessary for growers to introduce new technologies that integrate wireless sensor networks (WSNs) for crop monitoring and enable analysis of data collected by the WSNs [14].

In the last two decades, the advancement and innovation of science and technology in the sectors of telecommunications, electronics, software development, and AI have played a prominent role in what is now called SF [7]. 

### 2.2. Smart Farming (SF)

SF focuses on the application of good agricultural practices combined with the implementation of different emerging technologies, improving crop quality and production [15]. Some examples of applications are related to disease monitoring at planting [16] and control of crop variables [17,18,19]. Its objective is productive efficiency, yield, profitability, and mitigation of environmental impact through the implementation of different techniques such as intelligent irrigation and precision in the application of pesticides and fertilizers [20].

SF brings together different technological developments, including AI, big data (BD) [21], and IoT [22]. These help in the solution of the problems and challenges currently faced by the agricultural sector, especially the requirements of increased food production due to the growth of the world population [15], which, according to UN predictions, will reach 8.5 billion people in 2030, 9.7 billion in 2050, and 11.2 billion in 2100 [23]. Another challenge for SF is to reduce the digital divide in rural areas [24], which are generally correlated with low income levels. It is estimated that 40% of the world’s population lives in developing countries (e.g., Colombia and other Latin American countries) [25]. SF would drive important changes in social and economic structures, testifying to the rural population [26], and fostering private sector and government policies to strengthen efficiency and agro-industrial production. Another major challenge of SF is related to the efficient management of water, a vital resource in agriculture. This activity consumes 70% of the world’s freshwater, and 20% of the total cultivated areas are irrigated [27]. 

Most of the SF applications obtain information on physical variables in agricultural land, using IoT sensors that favor food production from collected data [6]. IoT allows monitoring of all processes involved in agro industrial production, automating control tasks for efficient production [28]. The massification of FS includes the joint work with telecommunications access networks that support the connections of a large number of sensor nodes. Generally, the captured data are analyzed by machine or deep learning algorithms, requiring stable connections, bandwidth with high speeds, and very low latencies that could not be supported by current third- and fourth-generation networks (3G and 4G) [2]. Therefore, fifth-generation (5G) networks promise to leverage the implementation of SF in population power demand.

### 2.3. 5G Usage Scenarios

**Figure 2:**
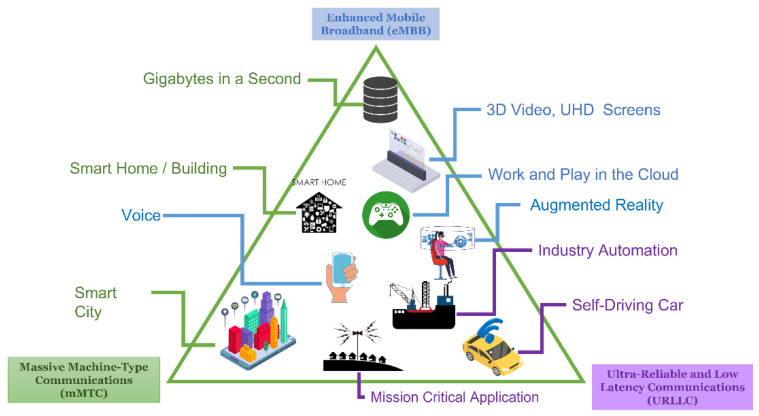
IMT scenarios for 2020 and beyond. Source: [7,29,30].

The International Telecommunication Union (ITU), in its recommendation ITU-R M.2083-0, defined the framework and general objectives for the future development of International Mobile Telecommunications (IMT) for the year 2020 and beyond, establishing the role of IMT in developed and developing countries to meet and enhance the future needs of an interconnected society. The authors of [7] summarized the role of IMT in three scenarios, as shown in Figure 2.

Figure 2 presents three scenarios defined by IMT: (1) enhanced mobile broadband (eMBB), focused on multimedia services consumption, (2) ultrahigh reliability and low latency (uRLLC), focused on industrial and emergency mission applications, and (3) massive machine type communications (mMTC), oriented to massive device connection (more than one million devices per km^2^). The above scenarios have different types of requirements, including high bandwidth, low latency, and capacity to support massive device connections. eMBB applications base their services on the cloud, virtual reality, and augmented reality, demanding greater network resources. On the other hand, uRLLC is in great demand for services and applications that require low latency and reliability in machine-to-machine (M2M) communications. Examples of uRLLC applications are autonomous cars, industrial communications, and intelligent electrical networks. In the case of the mMTC scenario, it is oriented to support the massive growth of connected devices, the product of IoT-based applications. Lastly, all these implementation scenarios are addressed in terms of the benefits of 5G.

### 2.4. 5G Frequency Bands and Their Applications

The “5G Spectrum” report presented by the Global Mobile Industry Association (GSMA) states that three key spectrum ranges are needed for 5G to support all use cases. The first corresponds to the frequency band below 1 GHz. This portion of the spectrum is an excellent option for extending 5G coverage in urban and rural areas since it has lower signal attenuation. In addition, the sub-GHz bands are a great support for the communication of IoT-based technologies. Some use cases of this band are found in Europe, where the 700 MHz band is used, and in the United States, where the 600 MHz band is used, both supporting 5G services. The next range (between 1 and 6 GHz) offers a very good ratio between coverage and capacity for 5G services. Some countries (e.g., Spain, Germany, and Japan) auctioned this frequency range to deploy 5G services. Lastly, frequencies above 6 GHz are necessary because they handle ultrahigh speeds provided by the available bandwidth, supporting uRLLC and enhanced broadband services [31]. Table 1 summarizes the frequency bands used in the agro-industrial sector and their main characteristics.

### 2.5. Projected 5G Frequency Bands in Colombia

Table 2 describes the status of the radio spectrum frequency bands in Colombia, including frequency bands, uplink, downlink, the status of use, availability, and operators that have been granted radio spectrum use permits. Regarding the assignment of operators, the Ministry of Information and Communications Technologies (MinTIC) issued nine resolutions assigning radio spectrum use permits for the 700 MHz and 2500 MHz bands to the following operators for the deployment of 4G telephony and internet services: Colombia Móvil S.A. (Tigo), Comunicación Celular. S.A Comcel.SA (Claro), and Partners (Wom).

**Figure 3:**
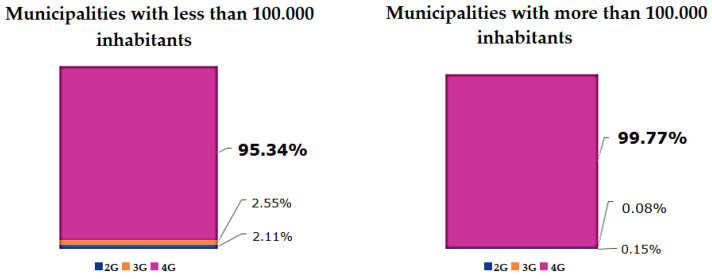
Coverage of 2G, 3G, and 4G technologies in Colombia. Source: [50].

Colombia currently uses the 700 MHz, 1900 MHz, and 2500 MHz bands with 2G, 3G, and 4G services. Up until the fourth quarter of 2021, 4G infrastructure deployment reached 95.34% coverage in municipalities with fewer than 100,000 inhabitants and 99.77% in municipalities with more than 100,000 inhabitants, as shown in Figure 3. However, it has been evidenced by MinTIC that, of the total number of active mobile telephony lines, 51% only use voice services without going beyond the navigation service, and that 21% of the total mobile internet accesses in the country are made through 2G and 3G technologies, especially in rural areas, limiting the features and benefits in terms of data transmission speeds offered by 4G [50].

This reality occurs despite the efforts made by MinTIC with the implementation of the Transition to New Technologies plan, and coverage expansion obligations undertaken by operators in the spectrum auction held in 2019 and defined in resolutions 3078 of 2019 and resolutions 330, 331, 332, and 333 of 2020. Regarding the 5G projection, MinTIC is betting on the 3500 MHz bands for the initial deployment. However, as of 2022, no spectrum auction has been held. Analog and digital TV broadcasting services currently occupy the 600 MHz band, but it is expected to be fully available in March 2029. As for the 26,000 MHz band, Colombia projects availability in 2027.

#### Relationship between IoT and 5G

IoT is becoming more popular and affordable every day, solving specific problems in different areas. Its mass deployment requires infrastructure that supports the connection of millions of devices, large bandwidths, and very low latencies [51]. These usage requirements cannot be efficiently met by 3G and 4G networks in a mass deployment scenario. Therefore, the advent of 5G is expected to meet these demands [52]. Certainly, 5G has great benefits in terms of transmission capacity, simultaneous connections, throughput, and security performance.

**Figure 4:**
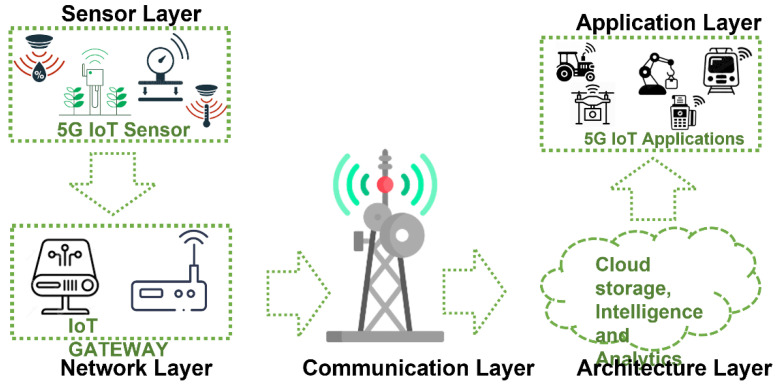
General 5G/IoT architecture. Source: [36].

Figure 4 shows the 5G/IoT architecture composed of five layers known as sensor layer, network layer, communication layer, architecture layer, and application layer. The first layer collects physical magnitude data from the sensors. This information is then processed, transmitted, and shared with other devices connected to the network. Finally, application-specific actions are taken [53].

### 2.6. Implementation of 5G Deployment in Colombia

In the case of Colombia, the MinTIC designed in December 2019 the 5G Plan that establishes the public policy guidelines and strategies that will serve as the basis for the deployment and massification of 5G in Colombia. This document contains the main challenges, strategies, and lines of action related to the radio spectrum, 5G pilots, the development of business models (applications and solutions) in 5G, digital security, and regulatory barriers to infrastructure deployment [54]. This process has been possible given the commitment of MinTIC, which through Resolution 638 of 2020 on 1 April 2020, initiated the granting of permits for the use of the radio spectrum to conduct pilot tests using 5G mobile technologies [55].

In compliance with the 5G Plan, on 9 March 2020, MinTIC published Resolution No. 467 which establishes the procedure for granting temporary permits for the use of the radio spectrum for technical tests [56]. Additionally, Resolution No. 638 of 1 April 2020 [57] opened the process of assigning permits for the use of the radio electric spectrum to carry out pilot tests using 5G mobile technologies through Resolution No. 722 of 30 April 2020 [58]. MinTIC granted permits to conduct pilot tests in the 3500 MHz frequency nationwide. The operators that were selected to carry out such tests in the cities of Bogotá, Bucaramanga, Cali, Medellín, and Tolú [59] are listed in Table 3.

#### Frequency Bands Identified in the 5G Deployment in Colombia

MinTIC, in harmony with the recommendations of the 2019 World Radiocommunication Conference, made available a set of frequencies enabled for the provision of 5G. The selection of these was based on the technical and commercial requirements demanded by 5G, as well as the interests of the nation. This section presents the current status of the aspiring frequency bands for 5G deployment in Colombia developed by the National Spectrum Agency (ANE) [54] (see Table 4).

According to the National Spectrum Agency (ANE), the 600 MHz band is considered fundamental in the Americas region for the upcoming 5G coverage deployments; thus, its occupation in Colombia is being reviewed, as is the availability of equipment in the market [49]. In addition, ANE is currently conducting three convenience studies to define technical parameters for frequency bands, guard bands, or other necessary technical measures to ensure interference-free operation of services operating in adjacency. The studies involve frequency blocks of 600 MHz, 900 MHz, and 2.3 GHz. Once these analyses have been completed, these parameters must be defined.

## 3. Materials and Methods

We detail the methodological route traced for the construction of the article in the aspects related to the search, analysis, and selection of information.

### 3.1. Search

The search process was divided into two stages. The first focused on the consultation and review of government documents, and the second focused on applying search strings in scientific databases. As for the search in government documents, the goals, projections, and documents concerning agriculture and 5G were consulted (see Table 5).

The second step supported by this article is a systematic literature review (SLR) in which open access articles indexed in the specialized databases of IEEE, Scopus, Science Direct, Springer, ACM, and Web of Science were extracted. In this case, search strings were applied using the words “smart farming”, “precision agriculture”, “agriculture”, “5G”, “IoT”, and “Colombia”. The documents consulted were published between 2016 and 2022.

### 3.2. Analysis 

Although the timeline for the selection of scientific articles was 6 years, those published after 2019 were analyzed, given that most of the definitions of 5G in terms of the spectrum were adopted at the World Radiocommunication Conference 2019—WRC-19. The selection of documents was made on the basis of the keywords mentioned above and included the following criteria: (1) theoretical elements related to the object of consultation; (2) elements that allow contextualizing the current situation of agriculture in Colombia; (3) 5G deployment in Colombia and its projection to enhance SF.

### 3.3. Selection of Information 

Following the search and analysis of the documents found, we proceeded to document the main findings associated with the current state of agriculture in Colombia, as well as a characterization of the crops with the greatest presence in the country. In addition, some future applications of 5G/IoT around SF in Colombia are proposed.

## 4. Analysis of Results

### 4.1. Agriculture and Applications Supported by the 5G Network

Currently, the agricultural industry is facing challenges to support farmers to take full advantage of new technologies, with 5G being one of the main innovations. According to expert considerations, 5G will be responsible for generating major changes in farm productivity [60]. In this context, innovation in agriculture emerges as a crucial priority and an essential component of the post-COVID-19 recovery.

**Figure 5:**
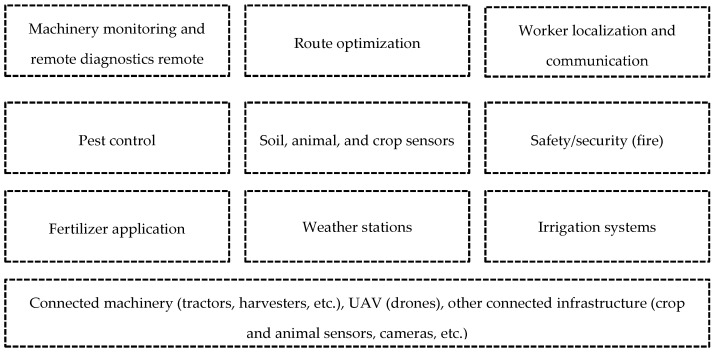
Use cases for agriculture [62].

It is expected that 5G-supported SF will enable the deployment of many sensors to measure and monitor in real time the state and quality of the soil, moisture, nutrients, etc., providing answers to various questions that will improve efficiency and generate savings in agricultural production costs, among other advantages [61]. A brief description of the main agricultural activities shows that there are opportunities for the use of 5G technology at every stage of production, including land preparation, planting, harvesting, and post-harvest. However, currently, the processes developed in the production stages are limited by the fact that data are collected manually (offline), making them difficult to analyze, plan, and make decisions about crops and animals. This and other limitations of the sector can be improved with the use of 5G [62], such as those listed in Figure 5. Table 6 presents a summary of the results of 5G applications in SF.

Although the benefits of 5G are numerous, many technical issues still need to be resolved to achieve an efficient deployment of this technology to ensure reliable data transmission in SF scenarios. Given that transmitted bits are susceptible to errors caused by phenomena such as noise, fading, or interference, and that the propagation of radio waves in the millimeter bands represents a real challenge from the point of view of coverage and data reliability, one of the challenges facing 5G is related to the optimization of channel coding and, consequently, error correction codes. In this regard, the replacement of traditional turbo codes with other techniques such as low-density parity check (LDPC) or polar codes has been considered. The former have better performance characteristics, but are difficult to implement due to their computational complexity, while polar codes are easier to implement thanks to less complex algorithms, but at the cost of sacrificing performance, especially at low coding rates [68,69].

Another challenge for 5G/IoT deployment in smart farms is the power consumption of user terminal equipment (UE), which are often located in rural or remote areas and, therefore, need to extend the charging time and lifetime of their batteries. In 5G, the cyclic prefix orthogonal frequency division multiplexing (CP-OFDM) waveform is used in the downlink, while CP-OFDM or discrete Fourier transform orthogonal frequency division multiplexing (DFT-OFDM) can be used in the downlink. Since the peak average power ratio is lower in DFT-OFDM, its adoption will decrease the power consumption in UEs, which is necessary for IoT systems in SF [70]. In addition, the possibility of using higher-frequency bands (e.g., above 70 GHz) in the future in 5G and the future 6G makes it necessary to consider single-carrier waveforms.

#### Agricultural Panorama in Colombia 

The agricultural sector has been a strategic actor in the country’s development, contributing to the increase in gross domestic product (GDP). However, this growth has historically been generated by the expansion of planted areas and not by increased productivity. Furthermore, in recent years, the sector has faced difficulties in terms of agricultural productivity, market access, and quality standards [71].

**Figure 6:**
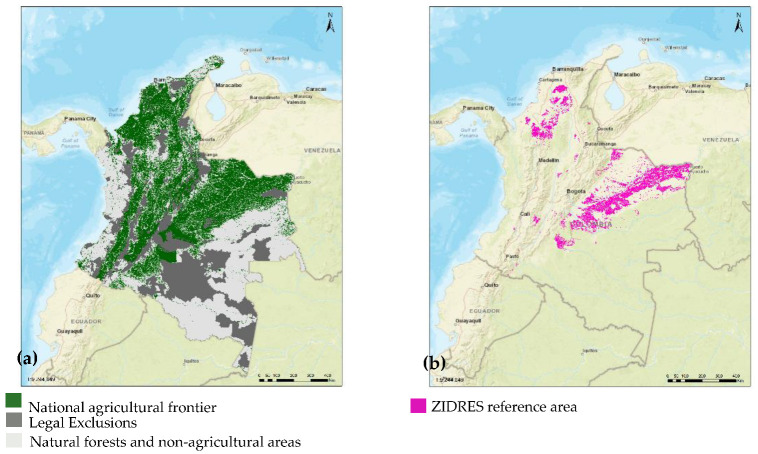
(**a**) Map of the agricultural frontier in Colombia (highlighted in green); (**b**) map of zones of interest for rural, economic, and social development (Zidres) in Colombia (highlighted in purple). Source: [72].

In Colombia, the growth of the agricultural sector has traditionally depended on the production of coffee and sugar, as well as on the expansion of other crops, such as fruit trees (avocado, pineapple, and cocoa) in recent years [71]. This growth has its frame of reference in the agricultural frontiers defined in Colombia, shown in Figure 6 and Table 6. These areas make it possible to separate the territory protected by Colombian law for various purposes, including (a) protected areas, (b) areas of special ecological importance, and (c) other areas where agricultural activities are permitted [72]. This distribution of soil availability for different agricultural uses (agroforestry, livestock, forestry, and conservation) reveals the main advantages of the country, since they are not concentrated in a single region but are dispersed throughout the territory; by having different thermal floors and diverse agroecological conditions, this allows the production of different goods from agriculture. This means that many of the country’s departments can produce a great diversity of products and that the same crop can be grown in distant areas that are geographically distant from each other.

According to official figures from the *Unidad de Planificación Rural Agropecuaria* (UPRA), an entity attached to the Ministry of Agriculture, Colombia has 114 million hectares, 23.2% of which are suitable for cultivation [73]. Undoubtedly, Colombia, taking advantage of its intertropical and equatorial location, has an important agricultural production potential capable of generating significant benefits from exports, as well as satisfying the food security of its population [74].

Despite having an approximate agricultural potential of 40 million hectares, Colombia only cultivates 19% of its land, presenting a lag in the agricultural production demanded by its population, especially that necessary to produce nutrient-balanced food [73]. Table 7 shows a summary of the areas for agricultural activity, according to each associated category.

**Figure 7:**
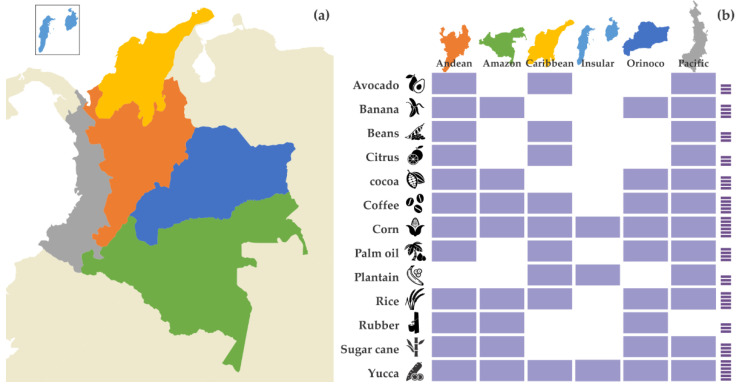
(**a**) Political map of Colombia’s regions; (**b**) agricultural products grown in most of the country’s regions.

In Colombia, rural, economic, and social development interest zones (Zidres) were created by Law 1776 of 2016 [75]. They are territories with agricultural, fish and forestry, and fish farming aptitude established from comprehensive rural development plans in a framework of the formal economy and land management, to promote competitiveness and insertion of human resources in a context of sustainable development, regional economic growth, social development, and environmental sustainability [76]. The diversity of crops associated with the soil characteristics of each department and its Colombian region are shown in Table 8, Table 9, Table 10, Table 11, Table 12, Table 13 and Table 14, and Figure 7 [77,78,79,80,81,82,83,84,85,86,87,88,89,90,91,92,93,94,95,96,97,98,99,100,101,102,103,104,105,106,107,108,109,110,111,112,113,114,115,116,117,118,119,120,121,122,123,124,125,126,127,128,129,130,131,132,133,134,135,136,137,138,139,140,141,142,143,144,145,146,147,148,149,150,151,152,153,154,155].

PA and SF applications that enhance agricultural production in Colombia can be increased and massified in rural areas, taking advantage mainly of the sub-gigahertz bands and the 5G mMTC scenario for internet access and a communication platform between nodes deployed in agricultural fields. In this sense, there are success stories from other countries in the world that serve as a reference, such as monitoring systems in orange crops in Brazil that record the temperature, humidity, and solar radiation in their crop fields, irrigation control systems in rice fields in China [156], and olive trees in Italy [157].

Regarding the Colombian case, several projects involving the use of IoT in agriculture are being developed [158]. One of the most important is the monitoring of agroclimatic variables, which uses data obtained as a tool to help in decision making and improve the management of crops. In [159], they implemented a remote terminal module that allows monitoring temperature, pH, oxygen level, and relative humidity in pineapple and Orellana mushroom crops located in the department of Casanare. In addition, the proposed system allows data transmission through 3G and 4G, so that it can be integrated with 5G in the future, taking advantage of its benefits. There are also similar PA pilots in tomato crops in greenhouses located in the department of Valle del Cauca, where they monitored air temperature, relative humidity, soil temperature, and soil moisture to find optimal ranges of these variables mitigating the effects of soil and climate change on the plantation [160].

The optimization of water use in irrigation systems is also widely studied in SF and PA projects, since, as mentioned above, agriculture is one of the activities that contribute most to its use and waste. Within the group of projects of this type in Colombia, there are applications in sugar cane crops in the department of Valle del Cauca [161] and squash crops in the department of Sucre [162]. In both cases, although with different techniques, IoT optimizes water resources. It should be noted that, in [163], the data transmission included a hybrid communications stage based on Zigbee and GPRS, UMTS, and HSDPA.

Other projects go beyond just monitoring and aim to fully implement SF. An advanced example is found in [163] with the implementation of a three-layer system: agricultural perception, edge, and data analysis in coffee plantations, which is one of the most important and traditional crops in Colombia. The functioning of the prototype was validated on a farm located in the department of Cauca. In addition to monitoring the variables, the proposed system can estimate the future production of the crop thanks to the use of ML (machine learning), allowing farmers to better plan the activities before and after the harvest. Furthermore, in [164], they created a complete tool that apart from monitoring allows farmers to use smartphones to get remote assistance. In addition, they created an app that allows estimating production and preparing it for the marketing process from the data entered by farmers.

Due to the large number of devices per square kilometer that will be required in SF, mMTC is the application scenario that will be best suited to use 5G in agricultural environments. In addition, given the large extension of many crops in Colombian territory (e.g., palm, rice, sugar cane, and coffee) located mainly in rural areas characterized by lower population, bands below 1 GHz become the best deployment option due to the lower attenuation they suffer concerning distance, allowing larger coverage areas. However, all these applications in real scenarios can only be carried out to the extent that the Colombian state and the different operators adopt the necessary strategies to offer 5G coverage in the most remote areas, thus reducing the digital divide.

## 5. Conclusions

Since the implementation of the Plan for the Transition to New Technologies and the spectrum auctions held in 2019, important 4G coverage figures have been reached such as 95.34% in municipalities with fewer than 100,000 inhabitants and 97.77% in populations with more than 100,000 inhabitants. However, there are challenges to mobile internet access, especially in rural areas that currently use 2G and 3G technologies, showing that efforts must continue to be made in synergy with mobile operators to take advantage of the installed capacity for 4G mobile internet access and continue with the deployment of infrastructure to reach 100%. With this scenario, it is expected that the Colombian government will soon authorize mobile telephony service providers to deploy and commercialize 5G, which will be beneficial for the development of applications oriented to SF.

A great opportunity is foreseen for Colombian agriculture with the projection of SF applications at every stage of production, from soil preparation to planting, harvesting, and post-harvest processes using tools such as BD, IoT, and AI that will be enhanced with the advent of 5G.

On the other hand, one of the main challenges that the country will have to face in rural and hard-to-reach areas is bridging the digital divide. Considering that 5G technology has a high initial infrastructure cost, its deployment will be concentrated in massively populated areas; therefore, extending it to rural areas will depend on the strategies contemplated in national and regional development plans, as well as public policies to promote increased connectivity and the number of 5G-based services. Likewise, since many farmers in Colombia are poor or do not have the means to invest in technological tools, it will be important for the state and operators to adopt economic policies that promote the acquisition of equipment and tools for this population to implement SF applications. In addition, it is necessary to implement technology training programs given the low educational levels in the rural areas farthest from the departmental capitals.

## Figures and Tables

**Table 1:** The 5G spectrum bands and their applications [32,33].

Band range	Typical Spectrum Types	5G App1	5G App2
<1 GHz	600 MHz, 700 MHz, 800 MHz, 900 MHz	Rural/unlicensed	Urban, WLAN(IoT)
1–6 GHz	1800 MHz 3.3–3.8 GHz	Urban/unlicensed	IoT/ITS
>6 GHz	(6–28 GHz)—24 GHz, 26 GHz, 28 GHz	UWB wireless fiber	Wireless VOD

**Table 2:** Status of 5G band projections in Colombia.

Band Range (MHz)	Total, Free Spectrum in Band (MHz)	Uplink (MHz)	Downlink (MHz)	Status	Operator	Reference
600	70	663–698	617–652	Available in March 2029	-	[34]
700	10	703–748	758–803	Before September 2022 (auction). For 4G use, most of this frequency band was licensed in December 2019.	Colombia Movil S.A (Tigo) (713–723 MHz couplet with 768–778 MHz) and (703–713 MHz couplet (758 MHz–768 MHz) Partners (Wom) 723–733 MHz couplet with (778–788 MHz. Comunicación Celular. S.A Comcel.SA (Claro) 733–743 MHz couplet with 788–798 MHz)	[35,36,37,38,39,40,41,42,43,44,45,46,47,48,49]
1900	5	1850–1910	1930–1990	For 4G use, most of this frequency band was licensed in December 2019.	Established for IMT with no regulations in force for its use.	[36,37,38,39,40,41]
2500	30	2500–2570	2620–2690	Before September 2022 (auction). For 4G use, most of this frequency band was licensed in December 2019.	Partners (Wom) 25154–(2520 MHz, couplet with 2635–2640 MHz) Comunicación Celular. S.A Comcel.SA (Claro) (2550–2555 MHz couplet with 2670–2675 MHz) (2545–2550 MHz 2665 couplet with 2670 MHz) (2540–2545 MHz 2660 couplet with 2665 MHz)	[37,38,39,40,41,42,43,44,45,46,47,48,49]
3500	400	3300–3700 (Canalization TDD)		Study auction	Unassigned operator	[34]
26,000	1000	24,250–27,500		Available in 2027	Unassigned operator	[31,49]

**Table 3:** Operators assigned for 5G pilot in Colombia.

No.	Operator	Frequency Bands
1	Colombia Telecomunicaciones S.A E.S.P	3500 MHz–3600 MHz
2	Comunicación Celular S.A Comcel S.A	3500 MHz–3600 MHz
3	Empresa de Telecomunicaciones de Bógota S.A E.S.P	3500 MHz–3600 MHz
4	ITICS S.A.S	3500 MHz–3600 MHz
5	Xiris Investment Group SAS	3300 MHz–3400 MHz

**Table 4:** Candidate frequency bands for 5G deployment in Colombia.

Frequency bands		Advantages	Disadvantages
Low band (frequencies below 1 GHz)	600 MHz band (614–698 MHz)	Lower propagation losses and, therefore, requires fewer base stations (BSs) to provide coverage	Lower available bandwidth compared to the rest of the bands
	700 MHz band (698–806 MHz)		
Medium bands (frequencies in the range of 1 to 6 GHz)	3.4 GHz band (3.3–3.4 GHz)	Spectrum harmonized with most countries in the world	High occupancy of the radio spectrum
	3.5 GHz band (3.4–3.6 GHz)		
	3.6 GHz band (3.6–3.7 GHz)		
High band (frequencies higher than 6 GHz)	Item A (24.25–27.5 GHz)	High amounts of radio spectrum are available for critical applications requiring low latency	High radio signal losses require a greater amount of BS to provide coverage
	Banda de 28 GHz (26.5–29.5 GHz)		
	Item B (31.8–33.4 GHz)		
	Item C (37–40.5 GHz)		
	Item K (71–76 GHz)		
	Item L (81–86 GHz)		

**Table 5:** Government documents consulted.

Subject Consulted	Colombian National Government Entity	Documents Consulted
Agriculture	National Development Plan (DNP) 2018 Departmental development plans, Agronet (Information and communication network for the Colombian agricultural sector).	National Development Plan (DNP) 2018–2022 “Pact for Colombia, Pact for Equity Agricultural and Rural Development Policy 2018–2022, agricultural frontier database
5G	National Spectrum Agency (ANE), MinTIC	5G Plan, New Technologies Transition Plan

**Table 6:** Summary of the results of 5G applications in SF.

Application	Technologies and Techniques Used	Results	Reference
Classification of fruit diseases	5G, IoT, neural networks	99.6% accuracy in the classification method, far superior to previously proposed techniques	[63]
Identification and tracking of livestock	5G, image recognition, blockchain (BC), BD, unmanned vehicles (UAV)	Reduction in labor costs	[64]
Security and surveillance of agricultural installations	5G, image recognition	90% accuracy in the identification of persons	[65]
Solving the problem of production control and efficiency in the agricultural food supply chain	5G, BC	The proposed system makes it possible to manage the crop from the sowing stage to the final marketing stage	[66]
Gathering information on the environmental, soil, and plant conditions of a cotton crop	5G, IoT, BD	10% increase in crop production	[67]
Control of the devices involved in crop management, plant growth management, and generation of notifications to users	5G, IoT, AI	Reduction in the costs of the agricultural process by using less equipment and labor	[5]

**Table 7:** Colombia’s agricultural frontier. Source: [72].

Category	Hectares	%
National agricultural frontier	39,600,142.6	34.71
Natural forests and nonagricultural areas	48,036,042.4	42.11
Legal exclusions	26,438,785.4	23.18

**Table 8:** Areas analyzed for the generation of areas in agricultural activity. Source: [72].

Activity/Coverage	Hectares	%
Agriculture	5,079,341	4.50
Grazing areas	31,156,166	27.30
Production forestry	135,235	23.18
Water bodies	1,704,041	0.10
Family farming	1,225,065	1.50
Nonagricultural	74,775,590	65.50
Total in the continental area	114,074,970	100.00

**Table 9:** Andean region.

Department	Cultivated Products	References
Antioquia	Coffee, banana, sugar cane, plantain, cocoa, corn, and rice	[77,78,79,80,81,82,83,84,85,86,87]
Boyacá	Potatoes, vegetables, cocoa, fruit trees, sugar cane, quinoa, and cereals	[77,79,80,82,84,88,89,90]
Caldas	Coffee, banana, sugar cane, avocado, citrus, and cocoa	[77,78,79,81,82,88,91,92]
Cundinamarca	Potatoes, carrots, tomatoes, onions, lettuce, corn, bananas, sugar cane, flowers, coffee, rice, and beans	[77,78,79,80,81,82,83,84,86,88,93]
Huila	Coffee, rice, banana, beans, maize, sugar cane, cocoa, and cassava	[77,78,79,81,84,88,94,95]
Norte de Santander	Coffee, cocoa, palm oil, sugar cane, banana, avocado, rice, and beans	[77,78,80,81,83,88,96,97]
Quindío	Banana, coffee, citrus, and avocado	[77,78,79,98,99,100]
Risaralda	Coffee, banana, avocado, sugar cane, corn, beans, tomato, onion, and various vegetables	[78,79,82,88,101,102]
Santander	Palm oil, cocoa, coffee, sugar cane, citrus, banana, rubber, pineapple, cassava, rice, corn, beans, tomato, onion, and several vegetables	[77,78,79,80,81,82,83,88,103,104,105,106]
Tolima	Coffee, rice, corn, banana, beans, sugar cane, avocado, cocoa, sugar cane, and mango	[68,69,70,71,72,73,78,107,109]

**Table 10:** Amazon region.

Department	Cultivated products	References
Amazonas	Yucca, banana, and various fruit trees	[79,110,111]
Caquetá	Banana, cassava, cocoa, rubber, coffee, sugar cane, rice, and maize	[77,78,80,88,112]
Guainía	Banana, cassava, maize, and cocoa	[78,79,113]
Guaviare	Maize, banana, cassava, rice, rubber, sugar cane, and cocoa	[79,83,114,115,116]
Putumayo	Cassava, corn, cocoa, sugar cane, chontaduro, pepper, cocoa, and banana	[77,78,79,83,90,117,118]
Vaupés	Cassava and cocoa	[119]

**Table 11:** Caribbean region.

Department	Cultivated products	References
Atlántico	Corn, cassava, mango, citrus fruits, pigeon peas, sorghum, and melon	[77,78,79,80,81,82,88,103,120,121,122]
Bolívar	Maize, oil palm, cassava, rice, yam, banana, cocoa, avocado, and plantain	[77,79,80,81,82,83,84,88,103,123,124,125]
Cesar	Palm oil, cassava, plantain, rice, corn, raisin, and melon	[77,79,88,80,108,109,126,127,128]
Córdoba	Corn, banana, cassava, rice, yams, cotton, oil palm, cocoa, etc.	[77,78,79,80,81,83,84,88,129,130]
La Guajira	Corn, coffee, cassava, rice, plantain, beans, oil palm, etc.	[77,80,81,103,83,131,132]
Magdalena	Palm oil, corn, cassava, yucca, banana, coffee, citrus, mango, and plantain	[77,79,80,88,133,134,135,136]
Sucre	Rice, cassava, corn, yam, plantain, oil palm, and pine	[77,78,83,84,103,137,138]

**Table 12:** Insular region.

Department	Cultivated products	References
San Andrés, Providencia y Santa Catalina	Coconut, yam, plantain, cassava, and corn	[79,103,139]

**Table 13:** Pacific region.

Department	Cultivated products	References
Cauca	Sugar cane, coffee, banana, corn, cassava, rice, cocoa, and coconut	[78,79,80,81,82,83,88,124,140,141]
Chocó	Banana, corn, rice, cocoa, and coconut.	[77,79,88,142]
Nariño	Coffee, potato, banana, cocoa, palm oil, sugar cane, pea, corn, coconut, and bean	[77,78,79,80,81,83,88,143,144,145]
Valle del Cauca	Sugar cane, coffee, banana, maize, citrus, banana, rice, pineapple, and avocado	[77,78,79,81,82,84,88,103,146,147]

**Table 14:** Orinoco region.

Department	Cultivated products	References
Arauca	Banana, cocoa, rice, corn, and cassava	[77,79,80,88,148,149]
Casanare	Rice, palm oil, coffee, corn, banana, and cassava	[77,78,79,80,83,88,150,151]
Meta	Palm oil, corn, sugar cane, rice, soybean, and banana	[77,81,85,90,152,153]
Vichada	Soybean, corn, palm oil, cashew, cassava, rice, rubber, and timber	[77,154,155]

## Data Availability

Not applicable.
